# ‘Green Zing’ and a selection of color concept descriptors on IQOS HEETS in Mexico

**DOI:** 10.18332/tid/155881

**Published:** 2022-11-18

**Authors:** Graziele Grilo, Joanna E. Cohen, Kevin Welding, Luz Myriam Reynales-Shigematsu, Maria Guadalupe Flores Escartin, Alena Madar, Katherine Clegg Smith

**Affiliations:** 1 Institute for Global Tobacco Control, Department of Health, Behavior and Society, Johns Hopkins Bloomberg School of Public Health, Baltimore, United States; 2 Departamento de Prevención y Control de Tabaquismo, Centro de Investigación en Salud Poblacional, Instituto Nacional de Salud Pública, Cuernavaca, Mexico; 3 Department of Health, Behavior and Society, Johns Hopkins Bloomberg School of Public Health, Baltimore, United States

**Keywords:** heated tobacco products, flavored tobacco, tobacco packaging, packaging policies

**Dear Editor,**

Flavored cigarettes, including flavor capsule cigarettes, are prominent in Mexico and in many other countries^1,2^. Flavors are communicated via colorful cigarette packs, flavor capsule imagery, flavor name (e.g. mentholated tobacco), usually in Spanish, and concept descriptors (e.g. ‘Mykonos Nightfall’), usually in English. Little is known about how flavor is communicated on packaging for other tobacco products in Mexico, such as sticks used with heated tobacco products (HTPs). Despite a presidential decree banning the importation of HTPs (including their sticks) in April 2021^3^, these products were available in the market. In May 2022, a second presidential decree also banned their sale and distribution^4^. We describe the availability and packaging characteristics of heated tobacco sticks in Mexico in October-November 2021.

The Tobacco Pack Surveillance System (TPackSS) systematically purchases unique tobacco packs (i.e. packs with at least one different exterior feature such as size or stick count) in selected countries, including Mexico^5^. Data collectors visited tobacco vendors (i.e. convenience stores, small/independent grocery stores, wholesalers, and pharmacies) in 12 low, middle, and high socioeconomic regions in each of five cities: Mexico City, Guadalajara, Leon, Durango, and Merida. Collectors used an observational checklist to note if heated tobacco stick packs were available for purchase and purchased any unique packs for the sample, which were then photographed. Photos were reviewed for the presence of flavor terminology/imagery, health warning label (HWL), and tax stamp.

HTPs were available in 5 of the 117 stores visited. Heated tobacco sticks were observed in stores in middle and in high socioeconomic regions in all cities, except Durango, and were sold for the same price of 63 MXN (100 Mexican pesos about 5.1 US$). Nine unique heated tobacco stick packs were purchased, all from the brand HEETS (Figure 1). All packs presented concept descriptors in English, written in different font colors. The country tax stamp and HWLs were present on all packs; while the color, content, and location of the front and back of the pack warnings corresponded to the Mexican HWL requirements, the side pack warning had different content despite having the required color and location^6^.

**Figure 1:**
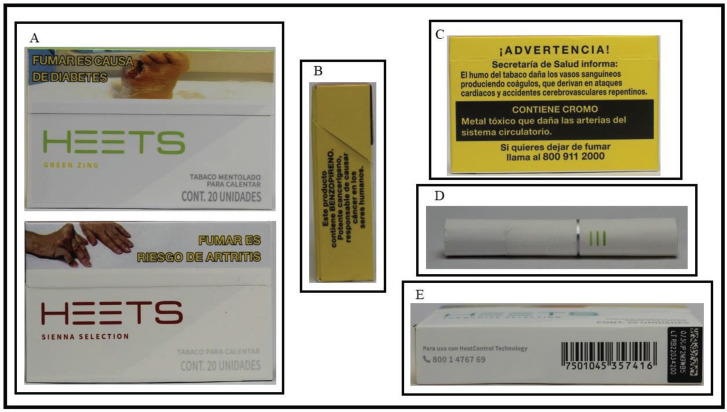
A) HEETS packaging – Green Zing contains the sentence ‘Mentholated tobacco to heat’ whereas Sienna Selection only says ‘Tobacco to heat’ (both phrases in Spanish). Besides Green Zing and Sienna Selection, the other concept descriptors used were: Purple Wave, Amber Selection, Russet Selection, Bronze Selection, Blue Selection, Turquoise Selection, and Yellow Selection. The packs include a Mexican pictogram (currently or previously required for cigarettes) placed at the top of the pack. B) Example of a side HWL – despite the colors and placement being in accordance with the regulation, the message is not. Packs contained one of the following messages, which are not the same currently or previously required for cigarette packs: ‘This product contains NICOTINE. Highly addictive chemical, and is the main reason why it is so difficult to quit smoking’, ‘This product contains NICOTINE. Nicotine is highly psycho addictive’, ‘This product is deadly, it contains CADMIUM. Toxic metal that inflames and damages lung tissue’, and ‘This product contains BENZOPIRENE. Potent carcinogen, responsible for causing cancer in humans’. C) Example of back Mexican HWL (all HEETS packs we purchased had a back warning from current or previous rotation required for cigarettes). D) Example of a heated tobacco stick. E) Mexican tax stamp on the right of the bottom of the pack (all HEETS packs we purchased had the tax stamp here). Images are available for public access on the Tobacco Pack Surveillance System (TPackSS) website (https://globaltobaccocontrol.org/tpackss/)

Similar to cigarette packs, HEETS packaging in Mexico communicates a flavor, taste, or sensation using concept descriptors and colors. Despite their low availability, the use of concept descriptors might appeal to new consumers, including youth^7^. Because HTPs and sticks are banned, Mexican packaging regulations do not apply to them; however, we observed a voluntary addition of a variation of Mexican HWLs on all packs, as reported in other circumstances^8,9^, which could possibly make the products appear legal. Whereas cigarette packs must have a side warning that is either ‘SMOKING CAN AGGRAVATE DAMAGE FROM COVID-19’ or ‘THIS PRODUCT IS ADDICTIVE’, we observed four different side messages on HEETS, none of which was a current or previous required side warning for cigarette packs. Our findings illustrate the use of similar tactics by the tobacco industry related to heated tobacco sticks as reported in Guatemala^10^; these findings can inform policymakers and tobacco control advocates about what to possibly expect with the introduction of HTPs in other markets.

## Data Availability

Data sharing is not applicable to this article as no new data were created.

## References

[1] Thrasher JF, Islam F, Barnoya J, Mejia R, Valenzuela MT, Chaloupka FJ (2017). Market share for flavour capsule cigarettes is quickly growing, especially in Latin America. Tob Control.

[2] Zavala-Arciniega L, Gutiérrez-Torres DS, Reynales-Shigematsu LM (2020). Prevalence, proportion and correlates of flavor capsule cigarette use in Mexico: results from the Ensanut 2018-19. Article in Spanish. Salud Publica Mex.

[3] Secretaría de Gobernación (2021). Decreto por el que se modifica la Tarifa de la Ley de los Impuestos Generales de Importación y de Exportación. In Spanish.

[4] Secretaría de Gobernación (2022). Decreto por el que se prohíbe la circulación y comercialización en el interior de la República, cualquiera que sea su procedencia, de los Sistemas Electrónicos de Administración de Nicotina, Sistemas Similares sin Nicotina, Sistemas Alternativos de Consumo de Nicotina, cigarrillos electrónicos y dispositivos vaporizadores con usos similares, así como las soluciones y mezclas utilizadas en dichos sistemas. In Spanish.

[5] Smith K, Washington C, Brown J (2015). The Tobacco Pack Surveillance System: A Protocol for Assessing Health Warning Compliance, Design Features, and Appeals of Tobacco Packs Sold in Low- and Middle-Income Countries. JMIR Public Health Surveill.

[6] Secretaría de Gobernación (2020). Acuerdo por el que se da a conocer la serie de leyendas, imágenes, pictogramas, mensajes sanitarios e información que deberá figurar en todos los paquetes de productos del tabaco y en todo empaquetado y etiquetado externo de los mismos, a partir del 1 de junio de 2020 y hasta el 30 de noviembre de 2021. In Spanish.

[7] Brown JL, Grilo G, Cohen JE (2022). Colours, capsules and concept flavour names on cigarette packs appeal to youth in Mexico. Tob Control.

[8] Wander N, Malone RE (2006). Making Big Tobacco Give In: You Lose, They Win. Am J Public Health.

[9] Hiilamo H, Crosbie E, Glantz SA (2014). The evolution of health warning labels on cigarette packs: the role of precedents, and tobacco industry strategies to block diffusion. Tob Control.

[10] Barnoya J, Monzon D, Pinetta J, Grilo G, Cohen JE (2021). New tobacco products, old advertising strategies: point-of-sale advertising in Guatemala. Tob Control.

